# Transcriptomic analyses reveal the underlying pro-malignant functions of PTHR1 for osteosarcoma via activation of Wnt and angiogenesis pathways

**DOI:** 10.1186/s13018-017-0664-2

**Published:** 2017-11-09

**Authors:** Shenglong Li, Yujin Dong, Ke Wang, Zhe Wang, Xiaojing Zhang

**Affiliations:** 1 Department of Bone and Soft Tissue Tumor Surgery, Cancer Hospital of China Medical University, Liaoning Cancer Hospital & Institute, 44, Xiaoheyan Road, Dadong District, Shenyang, Liaoning 110042 China; 2 Department of Hand and Foot Surgery, Dalian Municipal Central Hospital Affiliated of Dalian Medical University, Dalian, Liaoning 116033 China; 3 Molecular Pathology Testing Center, Foshan Chancheng Central Hospital, Foshan, Guangdong 528031 China; 4 Department of Orthopedics, Zhongshan Hospital Affiliated to Fudan University, Shanghai, 200032 China

**Keywords:** Osteosarcoma, PTHR1, Wnt pathway, Angiogenesis, Inflammation

## Abstract

**Background:**

Increasing evidence has indicated parathyroid hormone type 1 receptor (PTHR1) plays important roles for the development and progression of osteosarcoma (OS). However, its function mechanisms remain unclear. The goal of this study was to further illuminate the roles of PTHR1 in OS using microarray data.

**Methods:**

Microarray data were available from the Gene Expression Omnibus database under the accession number GSE46861, including six tumors from mice with PTHR1 knockdown (PTHR1.358) and six tumors from mice with control knockdown (Ren.1309). Differentially expressed genes (DEGs) between PTHR1.358 and Ren.1309 were identified using the LIMMA method, and then, protein–protein interaction (PPI) network was constructed using data from STRING database to screen crucial genes associated with PTHR1. KEGG pathway enrichment analysis was performed to investigate the underlying functions of DEGs using DAVID tool.

**Results:**

A total of 1163 genes were identified as DEGs, including 617 downregulated (Lef1, lymphoid enhancer-binding factor 1) and 546 upregulated genes (Dkk1, Dickkopf-related protein 1). KEGG enrichment analysis indicated upregulated DEGs were involved in Renin-angiotensin system (e.g., Agt, angiotensinogen) and Wnt signaling pathway (e.g., Dkk1), while downregulated DEGs participated in Basal cell carcinoma (e.g., Lef1). A PPI network (534 nodes and 2830 edges) was constructed, in which Agt gene was demonstrated to be the hub gene and its interactive genes (e.g., CCR3, CC chemokine receptor 3; and CCL9, chemokine CC chemokine ligand 9) were inflammation related.

**Conclusions:**

Our present study preliminarily reveals the pro-malignant effects of PTHR1 in OS cells may be mediated by activating Wnt, angiogenesis, and inflammation pathways via changing the expressions of the crucial enriched genes (Dkk1, Lef1, Agt-CCR3, and Agt-CCL9).

**Electronic supplementary material:**

The online version of this article (10.1186/s13018-017-0664-2) contains supplementary material, which is available to authorized users.

## Background

Osteosarcoma (OS) is the most frequent primary malignant bone tumor developed in the metaphyses of long bones during childhood and adolescence, with an estimated incidence of approximately 3.5 per million [1, 2]. Despite intensive multiagent chemotherapy and surgical resection have dramatically increased the 5-year survival rate to 70%, death still occurs in about 30% of patients with OS due to recurrence and metastasis (specially to the lung) [1, 2]. Thus, improving understanding of the mechanisms of OS progression and exploiting underlying strategies for malignancy suppression has justifiably attracted a great deal of attention.

Recently, accumulating evidence has indicated parathyroid hormone type 1 receptor (PTHR1), a G-protein-coupled receptor, may play important roles in the pathogenesis of OS. PTHR1 is found to be highly expressed in OS cells and tissues (especially in metastatic or relapsed samples) [3–7]. Over-expression of PHTR1 promotes proliferation, motility, and invasion of OS cells, which can be reversed by shRNA-mediated gene silencing [3, 7, 8]. Further studies suggest PHTR1 may exert the tumor-promoting effects through being activated by its ligands, including parathyroid hormone (PTH) and parathyroid hormone-related peptide (PTHrP) [9, 10]. Upon activation by PTH/PTHrP, PTHR1 induces the generation of cyclic AMP (cAMP) from ATP through adenylyl cyclase followed by the release of cAMP-dependent protein kinases [9–11]. Active protein kinases (PKA, PKC, or ERK) move to the nucleus and phosphorylates transcription factors, such as cAMP-response element-binding protein (CREB) and runt-related transcription factor 2 (Runx-2) which ultimately lead to the development of OS through regulating the expression their target genes (TGF-b1, transforming growth factor b1; CTGF, connective tissue growth factor; FGF-2, fibroblast growth factor; HAS2, HA-synthase-2 [3, 12, 13]). However, the functions of PTHR1 in OS remain not fully understood.

The goal of this study was to further illuminate the mechanisms of PTHR1 by analyzing the microarray data of OS [8]. Differentially expressed genes (DEGs) between OS tissues with and without PTHR1 knockdown were identified and then protein–protein interaction (PPI) network was constructed to screen crucial genes associated with PTHR1, which was not performed in the study of Ho et al. [8]. Our studies may provide new insights into the mechanisms of PTHR1 in OS and reveal some potential targets for treatment of OS.

## Methods

### Microarray data

The microarray data of OS were extracted from the Gene Expression Omnibus (GEO) database (http://www.ncbi.nlm.nih.gov/geo/) under the accession number GSE46861 [8], which contained six tumors with shRNA PTHR1 knockdown and six tumors with shRNA control knockdown. The tumor tissues were obtained from Balb/c nu/nu mice undergoing mouse OS80 cell line injection into the back flank and grown for 4 weeks. Mouse OS80 was transfected with either renilla luciferase shRNA control (Ren.1309) or a shRNA specific for PTHR1 (PTHR1.358). Thus, PTHR1.358 and Ren.13096 cell samples were used to descript these two groups in the following analysis.

### Data normalization and DEG identification

The raw data (CEL files) downloaded from the Affymetrix Mouse Gene 1.0 ST Array platform GPL6246 were preprocessed and normalized using the Robust Multichip Average (RMA) algorithm [14] as implemented in the Bioconductor R package (http://www.bioconductor.org/packages/release/bioc/html/affy.html). The DEGs between PTHR1.358 and Ren.13096 cell samples were identified using the Linear Models for Microarray data (LIMMA) method [15] in the Bioconductor R package (http://www.bioconductor.org/packages/release/bioc/html/limma.html). After the *t* test, the *p* value was corrected with the Benjamini-Hochberg (BH) algorithm [16]. Genes with an adjusted *p* < 0.05 and |logFC(fold change)| > 0.5 were considered differentially expressed.

### PPI network construction

To screen crucial genes associated with PTHR1, the DEGs were imported into the PPI data that were collected from acknowledged STRING 10.0 (Search Tool for the Retrieval of Interacting Genes; http://string-db.org/) database [17]. The PPIs with combined scores > 0.7 were selected to construct the PPI network which was visualized using Cytoscape software 2.8 (www.cytoscape.org/) [18]. Three topological properties, including degree [the number of interactions per node (protein)], betweenness (the number of shortest paths that pass through each node), and closeness centrality (the average length of the shortest paths to access all other proteins in the network) were calculated using the CytoNCA plugin in cytoscape software (http://apps.cytoscape.org/apps/cytonca) [19] to rank the nodes in the PPI network. In addition, the Molecular Complex Detection (MCODE) plugin of Cytoscape software was also employed to identify functionally related and highly interconnected clusters from the PPI network with a degree cutoff of 2, node score cutoff of 0.2, k-core of 2, and maximum depth of 100 (http://baderlab.org/Software/MCODE) [20]. Significant modules were identified with MCODE score ≥ 4 and nodes ≥ 6.

### Function enrichment analysis

Gene ontology (GO) and Kyoto encyclopedia of genes and genomes (KEGG) pathway enrichment analyses were performed to investigate the underlying functions of all DEGs and the DEGs in PPI network using The Database for Annotation, Visualization and Integrated Discovery (DAVID) 6.8 online tool (http://david.abcc.ncifcrf.gov). A modified Fisher Exact *p* value < 0.05 was chosen as the cutoff point for GO and KEGG analyses.

## Results

### Identification of DEGs

After data normalization, 1163 genes were identified as DEGs between PTHR1.358 and Ren.13096 cell samples based on the threshold of adjusted *p* < 0.05 and |logFC| > 0.5, including 617 downregulated (such as Lef1, lymphoid enhancer-binding factor 1) and 546 upregulated genes (such as Dkk1, Dickkopf-related protein 1) (Table 1). Furthermore, PTHR1 was also found to be significantly downregulated (logFC = − 0.6919, adjusted *p* value = 0.0002), indirectly demonstrating the knockdown model had been established successfully.

**Table 1:** Top 15 upregulated and downregulated genes differentially expressed between Pth1r knockout osteosarcoma cells and control

Expression	Gene_Symbol	LogFC	Adjusted *p* value
Upregulated	Grin2c	2.272	4.27E−06
	Ccnb3	3.243	4.27E−06
	Usp51	1.702	4.45E−06
	Slc1a3	1.443	6.34E−06
	Kcnk1	2.774	6.34E−06
	Bmp3	2.764	9.74E−06
	Rbm44	1.846	1.14E−05
	Plppr5	2.010	1.85E−05
	Phex	1.977	1.85E−05
	Nell1	2.368	2.00E−05
	AI593442	1.477	2.00E−05
	Ooep	0.828	2.00E−05
	Trpc6	1.628	2.00E−05
	Dkk1	1.730	2.00E−05
Downregulated	Lef1	− 1.677	2.90E−06
	Elf1	− 1.300	4.79E−06
	Jph1	− 1.674	5.47E−06
	Zfhx4	− 2.183	6.34E−06
	S1pr3	− 1.209	8.30E−06
	Slc20a2	− 1.031	9.06E−06
	Pdgfc	− 1.073	9.20E−06
	Wbp4	− 1.221	9.74E−06
	Robo1	− 1.536	1.12E−05
	Dgkh	− 1.183	1.12E−05
	Mpped2	− 1.873	1.14E−05
	Dnajc15	− 1.661	1.24E−05
	Atp10a	− 1.118	1.85E−05
	Slc6a15	− 2.051	1.85E−05

*FC* fold change, *adjusted p value* the *p* value was corrected with the Benjamini-Hochberg (BH) algorithm

### Function enrichment analysis

The above differential genes were subjected to the online tool DAVID for function enrichment analysis with the mouse genome as background and *p* < 0.05 as the cutoff point. As a result, 24 KEGG pathways were enriched for upregulated DEGs, including Renin-angiotensin system (e.g., Agt, angiotensinogen), Renin secretion (e.g., Agt), and Wnt signaling pathway (e.g., Dkk1), while 16 pathways were for downregulated DEGs, including basal cell carcinoma (e.g., Lef1) (Table 2).

**Table 2:** KEGG pathway enrichment of all DEGs and modules

DEGs	Expression	Term	Count	*p* value	Genes
All	Upregulated	mmu05150:Staphylococcus aureus infection	13	1.16E−08	C1qa, C3ar1, C1qb, Selp, Fcgr2b, C4b, Fcgr4, H2-aa, Cfd, H2-ea.-ps…
		mmu05152:Tuberculosis	16	1.64E−04	Mrc1, Cd209a, Tlr1, Fcgr4, Ctss, Tlr9, Fcgr3, Vdr, Fcgr2b, Mapk13…
		mmu03320:PPAR signaling pathway	10	4.65E−04	Lpl, Cd36, Cyp27a1, Pparg, Fabp4, Aqp7, Fabp7, Adipoq, Acsl6, Angptl4
		mmu04145:Phagosome	15	4.89E−04	Mrc1, H2-m9, Cd209a, Fcgr4, Ctss, Fcgr3, Cybb, Cd36, H2-m11, Fcgr2b…
		mmu04610:Complement and coagulation cascades	9	0.001	C1qa, C3ar1, C1qb, C4b, Cd59a, F3, F8, Cfd, C1qc
		mmu04620:Toll-like receptor signaling pathway	10	0.002	Cd86, Ccl3, Mapk13, Irf7, Tlr1, Il12a, Ticam2, Tlr7, Tlr8, Tlr9
		mmu04514:Cell adhesion molecules (CAMs)	13	0.002	Selp, H2-m9, Cadm1, Neo1, Ncam1, Siglec1, Cd86, H2-m11, H2-aa, Cd4…
		mmu04614:Renin-angiotensin system	6	0.003	Ace, Agtr1a, Agt, Prcp, Cpa3, Enpep
		mmu04080:Neuroactive ligand-receptor interaction	18	0.004	C3ar1, Thrb, Grik2, Lpar3, Vipr2, Ednra, P2ry13, Aplnr, P2ry6, Adrb2…
		mmu04924:Renin secretion	8	0.004	Ednra, Ace, Adrb2, Agtr1a, Agt, Pde1a, Pde3b, Cacna1d
		mmu05330:Allograft rejection	7	0.005	H2-m9, Cd86, H2-m11, Il12a, H2-aa, H2-t24, H2-ea.-ps
		mmu04978:Mineral absorption	6	0.006	Vdr, Atp1b1, Mt2, Cybrd1, Slc40a1, Trf
		mmu04940:Type I diabetes mellitus	7	0.009	H2-m9, Cd86, H2-m11, Il12a, H2-aa, H2-t24, H2-ea.-ps
		mmu05142:Chagas disease (American trypanosomiasis)	9	0.010	C1qa, C1qb, Ace, Ccl3, Mapk13, Il12a, Smad3, C1qc, Tlr9
		mmu05332:Graft-versus-host disease	6	0.017	H2-m9, Cd86, H2-m11, H2-aa, H2-t24, H2-ea.-ps
		mmu05133:Pertussis	7	0.020	C1qa, C1qb, C4b, Mapk13, Il12a, Ticam2, C1qc
		mmu04310:Wnt signaling pathway	10	0.021	Fzd9, Dkk1, Sfrp1, Sfrp2, Fzd3, Camk2b, Fzd5, Daam2, Lrp5, Fzd6
		mmu04060:Cytokine-cytokine receptor interaction	14	0.025	Il1r2, Il1r1, Ccl3, Osmr, Ccl8, Pf4, Ccl7, Tnfsf10, Ccr5, Cxcl13…
		mmu04960:Aldosterone-regulated sodium reabsorption	5	0.027	Atp1b1, Sgk1, Nr3c2, Igf1, Insr
		mmu04380:Osteoclast differentiation	9	0.029	Il1r1, Cybb, Fcgr2b, Mapk13, Pparg, Fcgr4, Fhl2, Trem2, Fcgr3
		mmu04612:Antigen processing and presentation	7	0.031	H2-m9, H2-m11, H2-aa, Cd4, Ctss, H2-t24, H2-ea.-ps
		mmu04640:Hematopoietic cell lineage	7	0.034	Il1r2, Il1r1, Cd36, Cd59a, Cd33, Csf3r, Cd4
		mmu05140:Leishmaniasis	6	0.037	Mapk13, Il12a, Fcgr4, H2-aa, H2-ea.-ps, Fcgr3
		mmu05144:Malaria	5	0.049	Selp, Cd36, Il12a, Thbs4, Tlr9
	Downregulated	mmu01130:Biosynthesis of antibiotics	22	3.33E−06	Cyp51, Ldhb, Shmt2, Msmo1, Pfkl, Hmgcr, Pafah2, Pgd, Pfkp, Fdps…
		mmu01100:Metabolic pathways	63	2.00E−04	Ldhb, Sgms2, Alg1, Hmgcr, Cyp2s1, Pgd, Cyp2j6, Pgam1, Lss, Hlcs…
		mmu00100:Steroid biosynthesis	6	2.40E−04	Cyp51, Msmo1, Sqle, Lss, Dhcr24, Fdft1
		mmu05412:Arrhythmogenic right ventricular cardiomyopathy (ARVC)	10	3.55E−04	Itga5, Lmna, Itga11, Cacnb2, Sgcd, Actn1, Gja1, Lef1, Cacnb3, Sgcb
		mmu00010:Glycolysis/Gluconeogenesis	9	0.001	Pgm2, Ldhb, Tpi1, Pfkl, Pfkp, Pgam1, Adh7, Aldh3b1, Eno1
		mmu00240:Pyrimidine metabolism	10	0.005	Pold4, Umps, Pole2, Pold1, Cda, Uck2, Dpyd, Polr3c, Nt5e, Polr2a
		mmu00230:Purine metabolism	13	0.011	Ak4, Polr3c, Pfas, Polr2a, Pgm2, Pold4, Pole2, Pde1c, Pold1, Pde5a…
		mmu05410:Hypertrophic cardiomyopathy (HCM)	8	0.012	Itga5, Tgfb3, Lmna, Itga11, Cacnb2, Sgcd, Cacnb3, Sgcb
		mmu00030:Pentose phosphate pathway	5	0.014	Pgm2, Pfkl, Pgd, Pfkp, Dera
		mmu05414:Dilated cardiomyopathy	8	0.015	Itga5, Tgfb3, Lmna, Itga11, Cacnb2, Sgcd, Cacnb3, Sgcb
		mmu00670:One carbon pool by folate	4	0.021	Mthfd1, Shmt2, Aldh1l1, Mthfd1l
		mmu00051:Fructose and mannose metabolism	5	0.021	Akr1b8, Tpi1, Pfkl, Pfkp, Pmm2
		mmu05217:Basal cell carcinoma	6	0.029	Wnt4, Fzd1, Lef1, Ptch2, Axin2, Gli3
		mmu01200:Carbon metabolism	9	0.030	Tpi1, Shmt2, Pfkl, Pgd, Phgdh, Pfkp, Esd, Pgam1, Eno1
		mmu00900:Terpenoid backbone biosynthesis	4	0.034	Hmgcr, Fdps, Hmgcs1, Idi1
		mmu01230:Biosynthesis of amino acids	7	0.036	Tpi1, Shmt2, Pfkl, Phgdh, Pfkp, Pgam1, Eno1
Module	1	mmu04062:Chemokine signaling pathway	6	1.70E−05	CCR5, CXCL13, GNAI1, CCR3, CCR2, CCL9
		mmu04080:Neuroactive ligand-receptor interaction	6	1.00E−04	C3AR1, APLNR, P2RY13, S1PR3, HTR1B, LPAR3
		mmu04060:Cytokine-cytokine receptor interaction	4	8.90E−03	CCR5, CXCL13, CCR3, CCR2
		mmu04024:cAMP signaling pathway	3	4.80E−02	HTR1B, GNAI1, HCAR1

*DEGs* differentially expressed genes

**Fig. 1:**
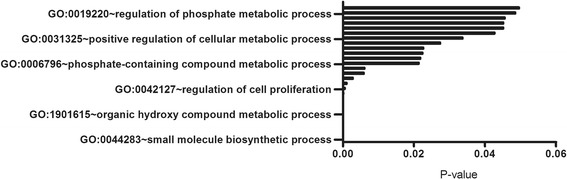
PTH1R enriched gene ontology (GO) terms for biological processes. Cell proliferation process was enriched

In addition, several GO terms, including 909 biological process (GO-BP), 63 cellular component (GO-CC), and 109 molecular function (GO-MF) categories were also enriched for upregulated DEGs, while 578 GO-BP, 42 GO-CC, and 48 GO-MF categories were for downregulated DEGs. To simplify the results, only the GO terms containing PTHR1 gene was displayed in this study (Fig. 1) because no KEGG pathway was obtained for PTHR1 gene. As expected, PTHR1 was found to be involved in cell proliferation process.

### PPI network construction

**Fig. 2:**
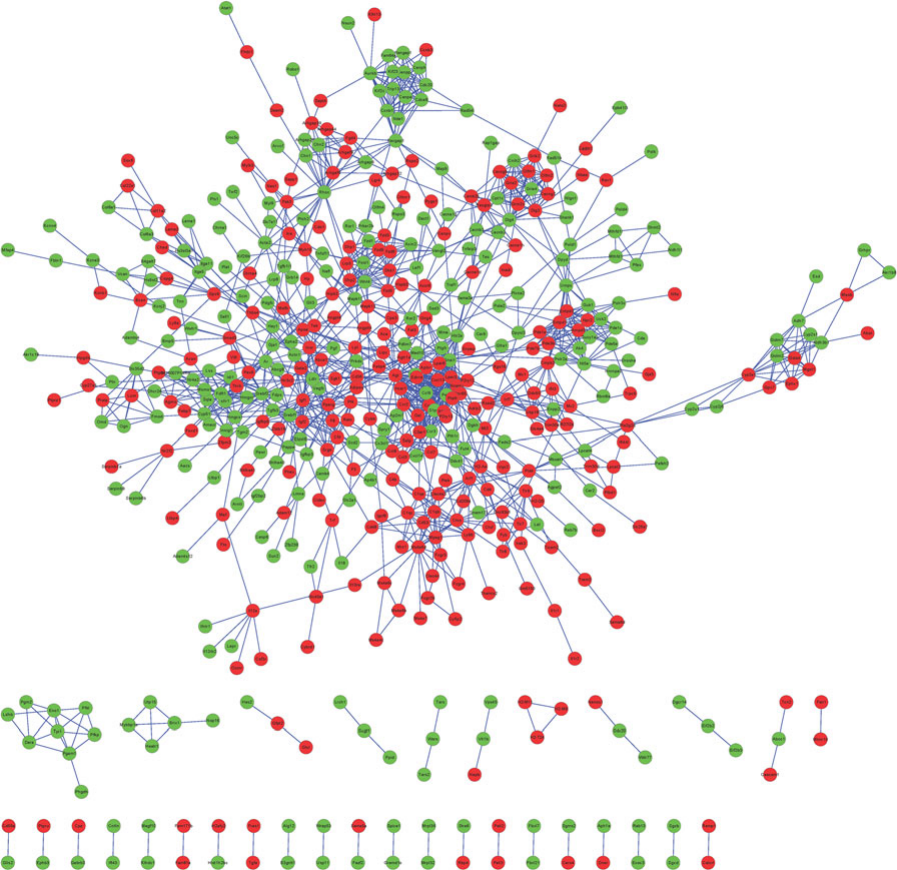
The protein–protein interaction network. The red and green nodes represent the upregulated and downregulated genes, respectively

**Fig. 3:**
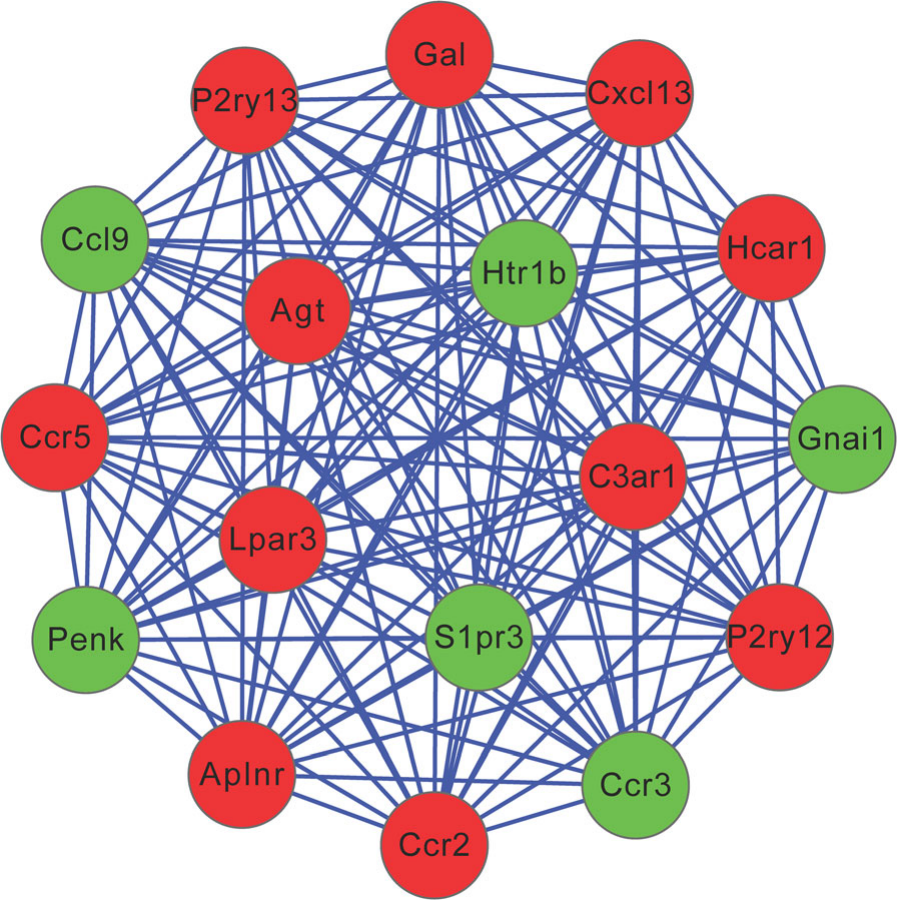
The most significant module extracted from protein–protein interaction network. The red and green nodes represent the upregulated and downregulated genes, respectively

A PPI network, including 534 nodes and 2830 edges (interaction relationships), was constructed after mapping the DEGs into the PPI data (Fig. 2; Additional file 1). By calculating the degree, betweenness, and closeness centrality, Agt gene was found to be the most key hub gene (Table 3). More interestingly, Agt was shown to interact with PTHR1 in PPI network, further indicating PTHR1 may promote the development of OS by influencing the expression of this gene. The importance of this gene was also confirmed in the module analysis (Fig. 3). Five modules were screened according to the given parameters (Table 4), among which module 1 (including Agt) was considered as the most significant with MCODE score = 8 and nodes = 17. Function enrichment analysis of module 1 (Table 2) showed chemokine- and cytokine-related inflammation pathways may be crucial, in which all enriched genes (CCR5, CXCL13, GNAI1, CCR3, CCR2, CCL9) could interact with Agt gene (Fig. 3; Additional file 1), indirectly illustrating the important role of Agt in OS.

**Table 3:** Hub genes in the protein–protein interaction network

Gene	Degree	Gene	Betweenness	Gene	Closeness
Agt	39	Agt	13,427.92	Agt	2.21E−05
Lpar3	26	Ar	8585.025	Srebf1	2.21E−05
Gnai1	25	Pla2g2d	8001.098	Ccr2	2.21E−05
Ccr5	24	Srebf1	7904.877	Ccr5	2.21E−05
Ccl9	24	Pax6	7664.532	Lpar3	2.21E−05
Ccr2	23	Rhoc	7643.579	Gnai1	2.21E−05
Ccr3	22	Dlg4	6747.256	Ptafr	2.21E−05
Igf1	21	Actn1	6420.109	Igf1	2.21E−05
Entpd1	20	Gnai1	6334.163	Ar	2.21E−05
Rhoc	20	Igf1	5806.686	Ccr3	2.20E−05
Dlg4	19	Racgap1	5675.621	Ccl9	2.20E−05
Entpd3	19	Cyp2e1	5666.956	P2ry12	2.20E−05
C3ar1	19	Lpar3	5255.704	Aplnr	2.20E−05
Ar	19	Gpc6	5126.2	C3ar1	2.20E−05
Dlg3	18	Tgfb3	5025.73	Htr2a	2.20E−05
P2ry12	17	H2-Aa	4732.818	Pparg	2.20E−05
Htr1b	17	Pld4	4646.952	Abca1	2.20E−05
Aplnr	17	Entpd1	4307.738	Cd36	2.20E−05
Srebf1	17	Htr2a	4269.31	Pf4	2.20E−05
Aurkb	16	Ctss	4234.501	Agtr1a	2.20E−05

*Degree* the number of interactions per node (protein), *betweenness* the number of shortest paths that pass through each node, *closeness centrality* the average length of the shortest paths to access all other proteins in the network

**Table 4:** Module analysis

Cluster	Score (Density × number of Nodes)	Nodes	Edges	Node IDs
1	8	17	136	Hcar1, Agt, Ccr2, Lpar3, Ccr5, Ccr3, Gnai1, P2ry12, P2ry13, S1pr3, C3ar1, Ccl9, Cxcl13, Aplnr, Penk, Gal, Htr1b
2	4.75	20	95	Cacng5, Agtr1a, Ednra, Lpar6, Gpr65, P2ry6, Ptafr, Ptgfr, Htr2a, Dlg4, Grin2c, Gria2, Grik2, Gria4, Dlg3, Cpt1c, Cacng7, Olfm1, Olfm2, Cnih2
3	4.667	12	56	Rangap1, Ccnb1, Cdc20, Kif23, Racgap1, Cdca8, Aurkb, Kif2c, Cenpe, Cenph, Cenpp, Nde1
4	4.4	10	44	Srebf2, Lss, Cyp51, Msmo1, Hmgcs1, Hmgcr, Sqle, Fdft1, Idi1, Fdps
5	4	9	36	Actn1, Igf1, Igf2, Vegfc, Tgfb3, Cfd, Pf4, Srgn, F8

## Discussion

Using the microarray data of OS provided by Ho et al. [8], we found PTHR1 knockdown could induce the upregulation of Dkk1, but the downregulation of Lef1. Dkk1 is thought to act as a soluble inhibitor for Wnt signaling [21], while transcription factor Lef1 mediates Wnt signaling pathway by binding with its co-activator β-catenin [22]. Several studies have demonstrated that activation of Wnt signaling promotes OS cell proliferation and invasion [23], but contrast results can be obtained after its inhibition [24, 25]. Accordingly, we believe Wnt pathway genes (Dkk1 and Lef1) may be an important downstream targets for PTHR1 to participate in the proliferation and invasion of OS, which was also identified in the study of Ho et al. [8]. Although accumulating evidence has confirmed the high expression of Lef1 regulates cell proliferation, migration, invasion, and cancer stem-like cell self-renewal, leading to poor prognosis of patients [26, 27], their roles in OS remain rarely reported, and thus, this gene may be a new target for further exploration. The role of Dkk1 in OS remains still controversial. In contrast to the theoretical expectation [28, 29], as well as our result (lower expression in OS), some scholars recently have identified the elevated expression of Dkk1 in OS tissues and cells [30, 31] and blockage of Dkk1 via a monoclonal antibody inhibits OS metastasis [32]. This indicates DKK1 represents a class of Janus-faced molecules with dichotomous roles in OS. We hypothesize the underlying mechanisms may be related with the status of p53 in OS. It has been reported that Dkk-1 can be induced by wild-type p53, but not by mutant p53 (R249S) [33]. Thus, the downregulation of p53 in OS with wild-type p53 may lead to the lower expression of Dkk-1, while Dkk-1 may be increased in a p53-independent manner for OS initiation and maintenance when p53 mutant occurs, which is similar to the regulatory mechanism between p53 and p21 in cancer [34]. Also, a recent study indicates exogenous introduction of p53 and Dkk1 could obviously inhibit the growth of OS cells, cause the cell cycle arrest at G0/G1 phase and apoptosis of OS cells compared with Dkk1 and p53 alone [35], further predicting a synergic relation between p53 and Dkk1. Zhang et al. further found the anti-proliferative effects of ursolic acid in OS cells may be mediated by upregulating p53 and then inhibit Wnt/β-catenin signaling [25]. In addition, p53 loss is observed to activate PTHrP-cAMP-CREB1 signaling [11] which is the downstream molecule of PTHR1 and thus may downregulate Dkk1 for OS as our study reported. However, further studies are also needed to confirm this mechanism of p53-PTHR1-Dkk1 in OS.

Furthermore, Agt was also shown to be upregulated after PTHR1 silencing. More interestingly, it could interact with PTHR1 in PPI network, indicating the change in its expression may be a crucial mechanism for explaining the roles of PTHR1 in OS, which was first identified in our study. Function enrichment analysis proved Agt may be involved in Renin-angiotensin system. As is well known, angiogenesis is an indispensable process for tumor growth and metastatic dissemination via providing essential oxygen and nutrients to proliferating cells and then a route for metastasis delivery [36]. Thus, targeted inhibition of angiogenesis may be potential approaches for prevention of OS progression. AGT, encoded by Agt gene, is a 452-amino-acid-residue protein that can be cleaved by renin to generate angiotensin I (AngI) which has been demonstrated to exert antiangiogenic properties in vitro and in vivo [37], suggesting the underlying anti-tumor activity of AGT. This conclusion has been further verified by recent studies. For example, Bouquet et al. showed adenovirus-mediated Agt overexpression inhibited tumor growth in preestablished human MDA-MB-231 mammary carcinomas in nude mice compared to controls and blocked tumorigenicity and pulmonary metastases of MDA-MB-231 and murine melanoma B16F10 cells when they were injected into C57BL/6 mice [38]. Vincent et al. revealed mice with bitransgenic HCC (hepatocellular carcinoma)/Hu-AGT-TG exhibited a significantly longer survival time than the HCC-TG mice and a decrease in both tumor growth and blood flow velocities of the liver through reducing of endothelial arterial markers (active Notch4, Delta-like 4 ligand and ephrin B2) [39]. However, the mechanism of Agt gene in OS remains unclear. In this study, we also predicted Agt might exert anti-malignancy effects by interacting with inflammation-related genes (such as CCR3, CC chemokine receptor 3; and CCL9, chemokine CC chemokine ligand 9). The relationship between inflammation genes and cancer development has been extensively studied. For example, it has been reported that CCR3 is highly expressed in breast cancer samples, especially luminal-like subtype [40]. Knockdown of CCR3 inhibited cellular proliferation, invasion, and migration, which was ERK signaling pathway-dependent [41, 42]. CCL9 was also shown to be highly induced in Gr-1 + CD11b + immature myeloid cells and premetastatic lung of tumor-bearing mice. Knockdown of CCL9 in myeloid cells reduced tumor cell proliferation and metastasis [43].

## Conclusion

Our present study preliminarily reveals PTHR1 may play important roles in the development and progression of OS by activating Wnt (Dkk1 and Lef1), angiogenesis, and inflammation pathways (Agt-CCR3 and Agt-CCL9). Lef1, Agt, CCR3, and CCL9 are all underlying new targets because no studies focused on them in OS. Thus, further in vitro and in vivo experimental studies were necessary to confirm the above findings. In addition, the role of Dkk1 is controversial and whether its expression is dependent on p53 status in OS also needs further investigation.

## Additional file

Additional file 1: PPI network construction. (TSV 148kb)

## Abbreviations

Agt: Angiotensinogen
AngI: Angiotensin I
BH: Benjamini-Hochberg
BP: Biological processes
CC: Cellular component
CCL9: Chemokine CC chemokine ligand 9
CCR3: CC chemokine receptor 3
CREB: cAMP-response element binding protein
CTGF: Connective tissue growth factor
DAVID: Database for Annotation, Visualization and Integrated Discovery
DEGs: Differentially expressed genes
Dkk1: Dickkopf-related protein 1
FGF-2: Fibroblast growth factor 2
GO: Gene Ontology
HAS2: HA-synthase-2
HCC: Hepatocellular carcinoma
KEGG: Kyoto encyclopedia of genes and genomes
Lef1: Lymphoid enhancer-binding factor 1
LIMMA: Linear Models for Microarray data
MCODE: Molecular Complex Detection
MF: Molecular function
OS: Osteosarcoma
PPI: Protein-protein interaction
PTH: Parathyroid hormone
PTHR1: Parathyroid hormone type 1 receptor
PTHrP: Parathyroid hormone-related peptide
RMA: Robust Multichip Average
Runx-2: Runt-related transcription factor 2
STRING: Search Tool for the Retrieval of Interacting Genes
TGF-b1: Transforming growth factor b1
